# Classification Muscles of the Horse

**Published:** 1883-01

**Authors:** 


CLASSIFICATION OF THE MOST IMPORTANT MUSCLES OF THE HORSE, with origin, insertion, nervous supply, and function of each. By E. Judson Peck, D.V.S.. A chart published by W. R. Jenkins.

